# Probabilistic Analysis of Pattern Formation in Monotonic Self-Assembly

**DOI:** 10.1371/journal.pone.0137982

**Published:** 2015-09-30

**Authors:** Tyler G. Moore, Max H. Garzon, Russell J. Deaton

**Affiliations:** 1 Department of Computer Science, University of Memphis, Memphis, TN, United States of America; 2 Department of Electrical Engineering and Computer Engineering, University of Memphis, Memphis, TN, United States of America; Massachusetts Institute Of Technology, UNITED STATES

## Abstract

Inspired by biological systems, self-assembly aims to construct complex structures. It functions through piece-wise, local interactions among component parts and has the potential to produce novel materials and devices at the nanoscale. Algorithmic self-assembly models the product of self-assembly as the output of some computational process, and attempts to control the process of assembly algorithmically. Though providing fundamental insights, these computational models have yet to fully account for the randomness that is inherent in experimental realizations, which tend to be based on trial and error methods. In order to develop a method of analysis that addresses experimental parameters, such as error and yield, this work focuses on the capability of assembly systems to produce a pre-determined set of target patterns, either accurately or perhaps only approximately. Self-assembly systems that assemble patterns that are similar to the targets in a significant percentage are “strong” assemblers. In addition, assemblers should predominantly produce target patterns, with a small percentage of errors or junk. These definitions approximate notions of yield and purity in chemistry and manufacturing. By combining these definitions, a criterion for efficient assembly is developed that can be used to compare the ability of different assembly systems to produce a given target set. Efficiency is a composite measure of the accuracy and purity of an assembler. Typical examples in algorithmic assembly are assessed in the context of these metrics. In addition to validating the method, they also provide some insight that might be used to guide experimentation. Finally, some general results are established that, for efficient assembly, imply that every target pattern is guaranteed to be assembled with a minimum common positive probability, regardless of its size, and that a trichotomy exists to characterize the global behavior of typical efficient, monotonic self-assembly systems in the literature.

## Introduction

Self-assembly is a process by which self-directed systems gain complexity over time through the local interactions of their simple components, and is a fundamental and pervasive process in natural phenomena including membrane formation and protein folding. In experimental self-assembly, systems are designed to execute an algorithm that is implemented through specific local interactions, for example, DNA template matching reactions. Study of the self-assembly of DNA-based nanostructures started with seminal work by Ned Seeman, Erik Winfree, Chad Mirkin, and others [1, 2]. DNA continues to be a promising material for construction of nanoscale structures and devices [3–11]. Other than DNA, diblock copolymers [12] and patchy particles [13] provide alternate mechanisms for algorithmic self-assembly. Although the results in this paper are potentially applicable to other systems, the focus here is on monotonic algorithmic self-assembly where stably bound components do not detach from the growing assembly.

The design of nanostructures and their experimental realizations have been mainly *ad hoc* or trial and error. In the lab, self-assembly is subject to incomplete reactions, binding errors, kinetic trapping, and other inevitable random effects. The result is that the desired, target structures are often lost in byproducts (junk) of the assembly process. Though revealing interesting capabilities of assembly systems, theoretical models have focused on the algorithmic power and properties of the *process* of assembly that are necessary to produce unique and exact target patterns. Therefore, there exists a disconnect between experimental practice, and theoretical models and results of algorithmic self-assembly.

In this paper, a probabilistic method of analysis to characterize how well self-assembly systems produce sets of target patterns is proposed. The method is not an attempt to model probabilistic mechanisms during growth, as in Chandran *et al*.’s probabilistic tile assembly model [14] or Cook *et al*.’s Markov chains [15]. Rather, it focuses on the relationship between the sets of intended target and actually assembled patterns. The targets are what the designer or experimentalist wants to be assembled, and the quality of an assembly system is measured by its ability to accurately construct the target set. Thus, strong assemblers should construct patterns that resemble target patterns, as well as produce a reasonable fraction of the target set. In addition, assemblers should make target patterns with a limited amount of impurities (or junk). Assembly systems that have both these properties are termed *efficient*. The strength and efficiency of assembly should depend not only on the assembly system, but also on the set of target patterns.

Probabilistic analysis is concerned with the overall behavior of pattern formation by the assembly system, regardless of the system’s primary components, model of interaction, or the particulars of the growth process. One can run a probabilistic analysis to compare the performance of two assembly systems on a given target set, even if one or both of them are deterministic, nondeterministic, and/or probabilistic. Although the approach suggested is quite general and applies to many types of assembly systems, the focus here is on algorithmic self-assembly systems that produce target patterns with a degree of nondeterminism. No particular assumption is made on the nature of this nondeterminism, which may range from errors due to experimental implementation of a model, such as stochastic or nondeterministic binding, to kinetic effects, or flexibility in the properties sought in the assembled patterns. The method might provide experimenters with a guide to choose assemblers that are more experimentally feasible. The method captures several notions of yield of an assembly process, as well as assemblers that are, borrowing from Valiant’s theory of the learnable [16], “probably approximately correct”.

An important issue in algorithmic self-assembly is the relative power of cooperative and noncooperative systems. In cooperative systems, aggregation of a single component to an assembly may require more than one binding event, as opposed to noncooperative systems in which a single binding event is sufficient. Cooperative systems are conjectured to be more computationally powerful than noncooperative ones, although noncooperative systems may be more experimentally feasible. A probabilistic analysis of several target sets of patterns that do not require cooperation [17] shows that noncooperative assembly systems are efficient. When cooperation is necessary to form target patterns, then, any degree of cooperation is more efficient than none at all. For example, in the formation of computational structures like binary counters, potential noncooperative binding results in assemblers that are not efficient. In addition, the requirements of efficiency impose limits on the uncontrolled accretion of components that may be addressed by constraining the assembly of patterns to finite areas or geometries that simulate experimentally accessible parameters, such as relatively small concentrations of reactants or small, fixed-size reaction vessels. Finally, some general results follow from the definitions of the method. For efficient assemblers, target patterns are assembled with positive probability independently of the dynamics of the assembler, and the dynamic behavior of a good assembler can be qualitatively characterized or estimated solely from the estimates of or bounds on its efficiency.

The layout of the paper is as follows. Some necessary background information is given in section 1. Section 2 provides precise definitions of the requisite concepts and discusses the precise characterizations of desirable properties such as “strength”, “purity”, and “efficiency”. Examples in section 3 are then presented of analyses of typical systems in self-assembly, some with respect to implementations under plausible error conditions. Section 3 then presents general results on probabilistic analysis concerning efficiency, as previously characterized. In the final section we provide some discussion and raise a few questions of interest arising from this approach to self-assembly.

## Background

The field of algorithmic self-assembly attempts to understand this process through models of molecular programs for the production of nanomaterials with complex structures, properties, and functionalities. Among the earliest and best known models is the abstract Tile Assembly Model (aTAM). Tile types are assigned four labels that abstract the bonding mechanism to the matching of labels or glues that color the sides of tiles [18, 19]. Growth of the assembly proceeds sequentially starting from some set of “seed” tiles. Tiles are attached to the growing assembly only if the glue strength of matching tile edges exceeds some designated “temperature” *τ*. Thus, temperature is an abstraction of the minimum energy required for bonding between tiles. Recently, an equivalent structure was formed by conjugating DNA oligonucleotides at right angles on the surface of a gold nanoparticle [20]. The model demonstrates that cooperative self-assembly processes operating at *τ* = 2 are at least as complex as arbitrary computation by programs equivalent to Turing machines. This property is referred to as computation universality [1, 21]. On the other hand, the assembly power of the model at temperature *τ* = 1 remains unknown [15], but is suspected not to be universal. From an experimental point of view, however, tile assembly at *τ* = 1 is considered to have advantages, such as speed and ease of implementation. Recent work has also proved that cooperative models are intrinsically universal [22], *i.e*., that there exist certain tile assembly systems that are capable of assembling simulations of any other tile assembly system in the model, up to a re-scaling factor.

The notation for the TAM is taken from [15, 18, 19, 23]. A tile type *t* is a “Wang” tile with glues (labels) *σ* ∈ Σ assigned to its 4 edges, *σ*_*N*_(*t*), *σ*_*E*_(*t*), *σ*_*S*_(*t*), *σ*_*W*_(*t*), corresponding to North, East, South, and West, from some alphabet Σ. A tile assembly system is a triple T = (*T*, *s*, *τ*), where *T* is a set of tile types, *s* ⊆ *T* is a set of seed or initial tiles, and *τ* is a temperature. In addition, there is the glue function *g*:Σ × Σ → ℤ^+^ ∪ 0 that evaluates the bonding strength of pairs of glues. An assembly *α* can then be described as a mapping from the integer lattice ℤ^2^ to *T* ∪ **empty**, where **empty** is a null tile with zero strength bonds to any adjacent tile type.

**Fig 1:**
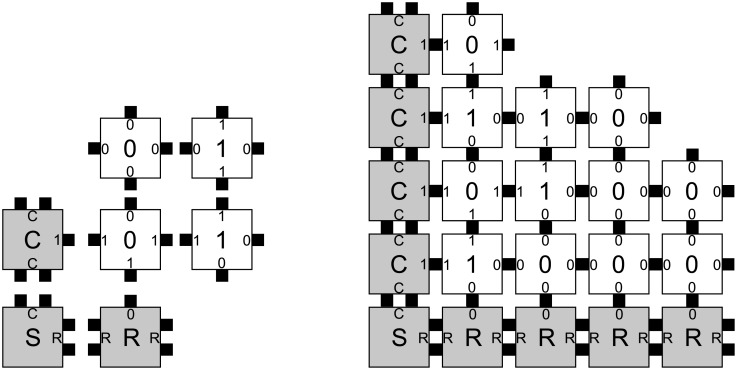
The binary counter tile set (Left) and a partial assembly (Right). The assembler counts from 0, represented as *n*-bits in the seed, by adding 1 to the previous layer up to the full *n*-bit value 2^*n*^ − 1.

Usually, tile self-assembly takes place on the 2D square lattice or grid, ℤ^2^, and temperatures are restricted to *τ* = 1 or 2. The assembly proceeds asynchronously and nondeterministically with new tiles *t* ∈ *T* attaching to eligible sites (*i*, *j*) ∈ ℤ^2^ if *α*_*i*, *j*_ = **empty** and *g*(*i*, *j*, *t*) ≥ *τ*. An assembly α ∈ **A**[𝒯] is terminal if no tile can be added that is stable at temperature *τ* (*τ*-stable). The set of terminal assemblies is **A**_◻_[T ] ⊆ **A**[T ]. An assembly sequence is called *locally deterministic* if each new tile binds with strength exactly *τ* to the existing assembly, each eligible site admits only one type of tile given that binding is allowed to occur only on pre-designated input labels on each tile type, and the the assembly sequence leads to a valid terminal assembly. A tile assembly system is *directed* if there is only one terminal assembly from any initial condition. If every assembly sequence associated with some tile assembly system is locally deterministic, then that tile assembly system is directed. An assembly sequence is a finite or infinite sequence of assemblies in which each assembly is a seed or is obtained from the previous one by the addition of one tile. An example of a binary counter implemented with a *τ* = 2 tile set is shown in Fig 1.

The family of tile assembly models includes a variety of other models, such as the 2-handed model or 2HAM [24] addressing unseeded arbitrary aggregation, the subsequent sTAM [25] in which tiles are given additional complexity and can react to local binding events, and the probabilistic pTAM [14]. Other models exist that account for the roles of kinetics [21], fuzzy temperature control [26], and global temperature scheduling and tile concentration programming [26–29] (see [30] for a survey). Other work has characterized the power of nondeterministic tile self-assembly to uniquely produce a single terminal assembly [31].

In addition to shedding light on the nature and complexity of the self-assembly process, tile assembly models are regularly used to analyze, predict and guide the results of molecular self-assembly in the laboratory. The kinetic Tile Assembly Model (kTAM) [21] is a nonmonotonic model of tile self-assembly that approximates the aTAM by controlling the forward rates (association) and reverse rates (dissociation) of tile types in the system. Other extensions of the aTAM allow for the robust and nearly optimal construction of a variety of unique terminal assemblies given a chance of error during aggregation using error-correcting and proofreading algorithms [32]. Despite this progress, tile assembly models have not fully addressed the role of errors in the assembly process, do not capture the intrinsic nondeterminism necessary for more realistic models or as seen in experimental nanofabrication, and have not completely explored the self-assembly power of other models, including noncooperative models of assembly [17, 33].

As mentioned above, it is important to distinguish the type of probabilistic analysis in this paper from probabilistic models of self-assembly, where tiles attach with some probability based on the local context of the attachment. For example, the pTAM [14] is designed to show that tile assembly sets smaller than the Kolmogorov complexity of the set of patterns being assembled are possible, at the price of producing some undesirable patterns, and gives estimates of the probability of 1D assemblies of a fixed length *N* being constructed with tile sets of cardinality *O*(*log* ^3^ *N*).

## Patterns and Assemblers

In this section we define a few key concepts associated with patterns and tile self-assembly. Informally, a pattern is a finite digital region of a two- or three-dimensional (2D/3D) integer lattice where each lattice point (*e.g*., a tile/cube) is labeled with a symbol from a fixed alphabet. For example, a black/white image is a pattern of symbols from a binary alphabet. The *support* of a pattern, sometimes called its *domain* or *shape*, is the underlying region in the lattice.

**Definition 1**. *A* pattern *x is a d-dimensional word over some alphabet* **∑** (*d > 0). The size of the pattern ∣x∣ is a nonnegative integer number associated with every pattern x, such as the number of unit squares/cubes in the corresponding region (its digital “area” or “volume”), the maximum Manhattan distance of a cell in the support from a designated origin (also called the* Manhattan radius *of the pattern), or the number of rows* (height) *or columns* (width) *in x*.

An example of a 2D pattern is shown in Fig 1. Ordinary words are defined up to rigid translations in the 1D integer lattice where two words that only differ by a translation are considered to be identical. The situation is slightly more complicated in two and higher dimensions, particularly in the context of tiling and self-assembly. An equivalent definition of size is a partition of the set of all possible patterns into layers, each consisting of all patterns of a particular size *n* ≥ 0, regardless of the concept of size being used. We partition the set of patterns by size and, consistent with the aTAM, assume that patterns of size *n* are obtained from patterns of size *n* − 1 or *n* by adding some additional labeled units, so that patterns of size at most *n* properly include those of size at most *n* − 1, for every *n* ≥ 0. In addition, patterns will be assumed to snugly fit to the coordinate axes, *i.e*., their support must contain at least one unit square on each axis and they are entirely contained in the first quadrant/octant.

The object of study is a pattern assembler defined as any experimental process, mechanism or logical model that attempts to generate certain patterns from some initial conditions, possibly acting nondeterministically. Much attention has been given to analyzing the capabilities of the aTAM and other tiling models by designing assemblers that simulate deterministic computations in order to ensure that the appropriate patterns will be error-free in the model. This work will focus on assemblers that will generally not produce patterns deterministically, or, even if deterministically, will produce patterns with a common set of features that characterize a possibly infinite family of target patterns. In general, the properties sought in the patterns of the target set may be dispersed throughout the patterns. Potentially desirable bulk properties of interest might include strength, conductivity, opacity, reflectivity, or plasmonic response. Other target properties include clusters of adjacent nonidentical labels (colorability), a ratio of one label to another (color mixing), and percolating subpatterns of active labels as discussed in Appendix A.

## Probabilistic Analysis

**Fig 2:**
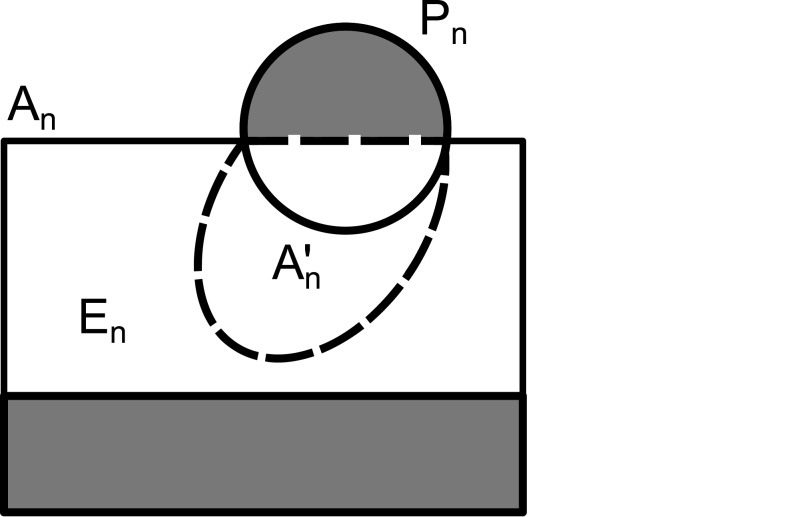
The three characteristic sets of assemblies in an assembly system. The set of assembled patterns **A**_*n*_ contains some target patterns (**A**_*n*_∩**P**_*n*_) that are already assembled, the set of promising patterns $A_{n}^{\prime }$ that includes **A**_*n*_∩**P**_*n*_ as well as nontarget patterns that will eventually become target patterns of larger size *n*, and the remaining error assemblies ($E_{n}=A_{n}-A_{n}^{\prime }$.)

This section addresses the primary question: What is the appropriate definition of assembly of a target set of patterns **P** given that the assembler G may produce a set of patterns **A** that potentially contains nontarget (or junk) patterns not in **P**? These two sets of interest are ideally the same, but in practice they may not be (Fig 2). At first, one might be tempted to require only that G produce a positive fraction of all patterns of every given size *n*, *i.e*., to impose the condition that for some fraction *p* > 0,

$$\forall n\;\;|A_{n}\cap P_{n}\left|\geq p\right|P_{n}|\;,$$ (1)

where **A**_*n*_ and **P**_*n*_ denote the set of patterns of size *n* in **A** and **P**, respectively. A set of patterns **P** is weakly probabilistically assemblable if this condition holds for some assembler G and some *p* > 0. Thus, when an assembler G weakly probabilistically assembles **P** with probability *p* = 1, G must assemble all the target patterns of every size *n*. In the aTAM, tile assembly systems are usually designed to implement an algorithm that produces the target set **P** with probability *p* = 1 because the patterns assembled are all in **P** and all the target patterns are eventually generated. In this case, **A** = **P**. In general, however, Condition Eq (1) is too weak to impose a hard constraint. For example, an assembler that produces every possible pattern of a given dimension weakly assembles any target set of patterns **P** with probability *p* = 1, since in that case **A**_*n*_∩**P**_*n*_ = **P**_*n*_. Yet, the assembler has no idea what the set of target patterns is, so it will generally produce an inordinate number of patterns not in **P** and cannot be considered “high yield” or “efficient”. We also note that if one begins with any given assembler, it will likewise weakly assemble the full set of patterns it produces with *p* = 1 if the target set is fixed to be that set *a posteriori*. In contrast, an experimentalist wishing to self-assemble a product often has a particular set of target patterns in mind before proceeding to find an appropriate process in the lab to produce them. More desirable assemblers are expected not only to produce a significant fraction of the target set, but also to have some “idea” of what the target set of patterns is like. Therefore, we also require that no pattern produced be significantly different from all patterns of the same size in the target set. Such a concept can be obtained by adding a second condition as in Definition 3.

### Strong and Efficient Probabilistic Pattern Formation

In order to evaluate the quality of an assembler, it is necessary to have a metric to quantify pattern similarity.

**Definition 2**. *A pattern z is a subpattern of a given pattern x if its support is fully contained in the support of x (after an appropriate shift) and their tile labels match cell-wise. Given a fraction 0 < p ≤ 1, two patterns x and y of identical size n are said to be congruent modulo p, denoted x ∼_p_y, if and only if both contain a common subpattern z of size at least p × n (after an appropriate shift.)*

Two patterns are thus congruent modulo *p* = 1 if and only if they are identical. They are congruent mod $\frac{1}{2}$ if they can be overlapped so that the labels of at least half of their unit cells match. Since every pair of patterns is congruent modulo 0, we assume that *p* > 0.

**Definition 3**. *A pattern assembler* G *probabilistically assembles a given target set of patterns* **P** *with respect to a notion of size n if and only if there exists a constant p > 0 such that*

$$\forall \;n\;\;|A_{n}\cap P_{n}\left|\geq p\right|P_{n}|,$$

*and*

$$\forall \;n\;\;\forall \;y\in A_{n}\;\;\exists \;\;x\in P_{n}\;\text{such}\;\text{that}\;x{∼}_{p}y,$$

*where* **A**_n_ (**P**_n_, *respectively) denotes the set of patterns of size n assembled by* G (*contained in* **P**, *respectively.) A set of patterns* **P** *is probabilistically assemblable if it is assembled with some probability p > 0 by some assembler* G . *If so, the value p is then the strength of the assembler*.

Informally, a (strong) probabilistic assembler has a good idea of the target set of patterns because every individual pattern it assembles captures a significant fraction *p* of some target pattern in the target set and because it captures a large fraction *p* of target patterns. A tight *p* can be regarded as the best common probability, across all sizes, of assembling a target pattern as compared to other target patterns. This value is different from the probability of assembling a target pattern with respect to the set of all assemblies, a matter that will be addressed in the next section. Note that an assembler G probabilistically assembles a target set **P** with *p* = 1 only when every pattern assembled is a target pattern and every target pattern is assembled, *i.e*. if and only if **A** = **P**. Thus we recover in this extreme case the usual notion of assembly used in the tile self-assembly literature, when restricting target patterns to terminal assemblies excluding their partial assemblies. For example, aTAM assemblers are designed to produce the algorithmically assemblable target set **P** with probability *p* = 1 because the patterns assembled are all in **P** and all the target patterns are eventually generated so that **A** = **P**. However, implementations of such systems for assemblers run *in vitro* usually do so with *p* < 1 for large target sets. Either some large fraction of patterns in the target set are not assembled perfectly, or the assembler produces some patterns that do not match closely with any pattern in the target set. Thus if **P** is regarded as the theoretically ideal stoichiometric yield, then, *p* can be regarded as an abstraction of the chemical reaction notion of “*yield*.”

In order to address the production of undesirable (or “junk”) patterns outside the target set **P**, a bound is placed on the fraction of nontarget patterns assembled. The set of assembled patterns of every size *n* is comprised of three characteristic subsets, namely the target patterns already assembled (**A**_*n*_∩**P**_*n*_), the promising patterns that will eventually become targets with further assembly ($A_{n}^{\prime }-\left(A_{n}\cap P_{n}\right)$), and the erroneous or junk patterns ($A_{n}-A_{n}^{\prime }$) that never will (Fig 2).

**Definition 4**. *Given a pattern assembler* G *for a set of target patterns* **P**, *an assembled pattern of size n > 0 is called a promising pattern if it is either a target pattern itself or it will eventually become a target pattern by valid further assembly on it as a seed. The set of these patterns is denoted* $A_{n}^{\prime }$. *Otherwise it is nonpromising, erroneous or an assembly error*. **E**_*n*_ *denotes the set of erroneous assemblies*.

For every size *n*, the set of erroneous patterns $E_{n}=A_{n}-A_{n}^{\prime }$ will never become targets and are thus “junk” produced by an assembler relative to the set of target patterns. It is clear that recognizing the set of promising patterns associated with some pattern assembler is, in general, not recursively solvable even in the aTAM. Often however, for physical systems, the concern is not just with the system’s ability to produce target patterns, but also with its ability to guarantee that the number of unavoidable error patterns produced by the assembly process is small relative to the target product.

**Definition 5**. *A set of patterns* **P** *is called* assemblable with impurity *q if there exists some assembler* G *and constant q > 0 such that*

$$\forall \;n\geq 0,\;\;|E_{n}\left|=\right|A_{n}-A_{n}^{\prime }\left|\leq q\right|P_{n}|\;\;$$

*If* **P** *is both probabilistically assemblable by an assembler* G *with strength p and impurity q, the inverse* $\frac{1}{q}$ *is called the purity and the ratio* $E=\frac{p}{q}$ *is called the* efficiency *of* G . *The set* **P** *is* efficiently probabilistically assemblable *if it is assemblable by some assembler with efficiency* $E=\frac{p}{q}>1$.

A tight such *q* characterizes how conservative the assembler is in forming patterns in the target set by bounding the size of the set of error patterns assembled with respect to the size of the target set. It can be regarded as the worst-case fraction, across all sizes, of assembled error patterns in relation to the set of target patterns. As mentioned above, efficiency metrics allow the comparison of the relative efficiency of two assemblers to produce a given target set.

Strictly speaking, Definitions 3–5 require a proper concept of assembler. However, we will abstain from a general definition because our analysis specifically applies to a number of well-known models and experimental techniques in nanoscale self-assembly, as described below. Moreover, we believe the framework is adaptable to other models not discussed herein. Although the definitions apply to assemblers in models that are nonmonotonic (such as the aTAM with negative strength glues [34]), the analyses become more complex, and so this work is mostly concerned with monotonic systems where tile detachment is not allowed.

### Examples

Next, several examples demonstrate the method of probabilistic analysis. Like complexity analysis of algorithms, probabilistic analysis of an assembler requires that a target to be assembled is chosen first, as well as a concept of pattern size *n*. The analysis requires estimation of the *p*’s and *q*’s, *i.e*., the worst-case probability of assembly strength and impurity in the assembled patterns, across all possible sizes *n*. The relative performance of different assemblers to realize the target set can then be compared using these measures. In addition, to investigate the effect of errors, we examine the extreme cases of perfect cooperativity or none.

While there exists target patterns that cannot be assembled by any noncooperative aTAM assembler [35], the cooperative aTAM is a powerful model for the algorithmic assembly of unique patterns. We begin with an analysis of the canonical cooperative aTAM assembler ℬ_*c*_, a binary counter [23]. Fig 1 shows the tile set and a partial assembly. The target set consists of 2D patterns producible from a single seed (representing 0 written in *n* bits) and enumerating in increasing order all binary numbers with up to the same number of bits. Thus, the target set is infinite, ∣*P*_*n*_∣ > 0 for all *n* > 0. The size of a pattern is the Manhattan radius of the pattern’s support taken from the seed at the origin (lower left corner.) Cooperative aTAM assemblers are capable of universal computation [21] and counting requires only the operation of addition by one (a local operation.) Therefore, assembly system ℬ_*c*_ is capable of producing this target set algorithmically. First, by some valid assembly sequences, ℬ_*c*_ is capable of assembling every pattern in the target set; therefore, **A**∩**P** = **P**. Second, patterns of Manhattan radius *n* assembled by ℬ_*c*_ match perfectly with at least one pattern of radius *n* in the target set, since ℬ_*c*_ correctly assembles the patterns of partial binary numbers and the target set includes all such patterns of the same radius. Therefore ℬ_*c*_ probabilistically assembles **P** with probability *p* = 1. We analyze the impurity of this assembler by first counting the size of $A_{n}-A_{n}^{\prime }$, *i.e*. we compute the number of junk patterns assembled by ℬ_*c*_. In this case, $|A_{n}-A_{n}^{\prime }|=0$. We again refer to the algorithmic construction and note that no tile can be placed in error in the model. Therefore, ℬ_*c*_ probabilistically assembles **P** with perfect purity (any *q* > 0 will do) and arbitrarily large efficiency *E*.

Now let us consider an assembler ℬ_*e*_ identical to ℬ_*c*_ except that it is run at temperature *τ* = 1. Tiles can now attach if only one glue matches an open edge, so the assembly is noncooperative. Thus, in contrast with the ideal ℬ_*c*_ at *τ* = 2, this case of *τ* = 1 represents the other extreme of error-prone assembly. Although the targets are still assembled weakly with *p* = 1, many other nontarget patterns are now assembled that can have an arbitrarily small number of cells in common with any target pattern of the same size. For each larger size, the proportion of common tiles decreases to arbitrarily small values as the number of bits and radius increase. Therefore, the strength of the assembler cannot be any positive *p* > 0. Likewise, an arbitrarily large fraction of assembled patterns of size *n* are not target patterns, so that the impurity *q* also deteriorates (requiring larger and larger values for *q*.) Therefore, because the junk grows much faster than the target patterns as *n* increases, the assembler effectively has efficiency *E* = 0. We remark that similar probabilistic analyses are obtained for other systems deterministically producing unique shapes or finite sets of patterns, such as squares [18].

**Fig 3:**
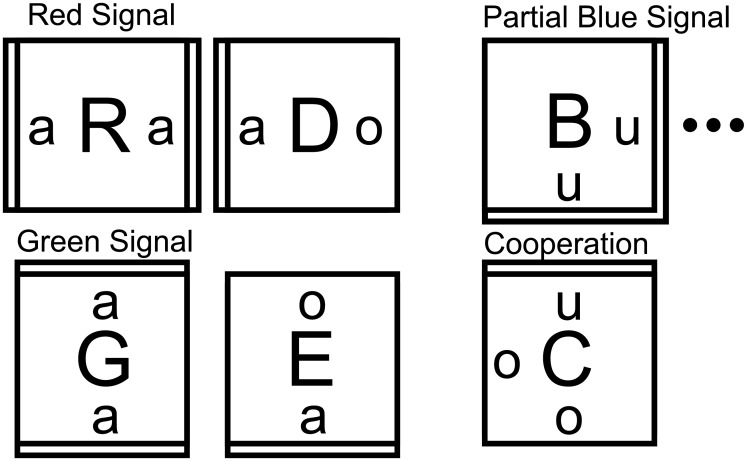
A tile set designed for a temperature 2 assembly system that uses cooperation to simultaneously detect nondeterministic input signals, red (R) and green (G), and from the designed point of cooperation output a deterministic signal to the blue (B) site. For cooperative assembly, double edges indicate strength 2 glues, and single edges strength 1. When the system is run in noncooperative mode, all glues are strength 1. R and G propagate from the inputs r and g by a series of R and G tiles. The D and E tiles are placed nondeterministically at some point along the signal to enable attachment of the cooperation tile C. At the point of cooperation, the O tile binds cooperatively, and then, grows the deterministic blue signal by a series of B tiles (only the first one is shown here.) The number of tiles in the blue signal is constrained by the size of the assembly space *L*.

**Fig 4:**
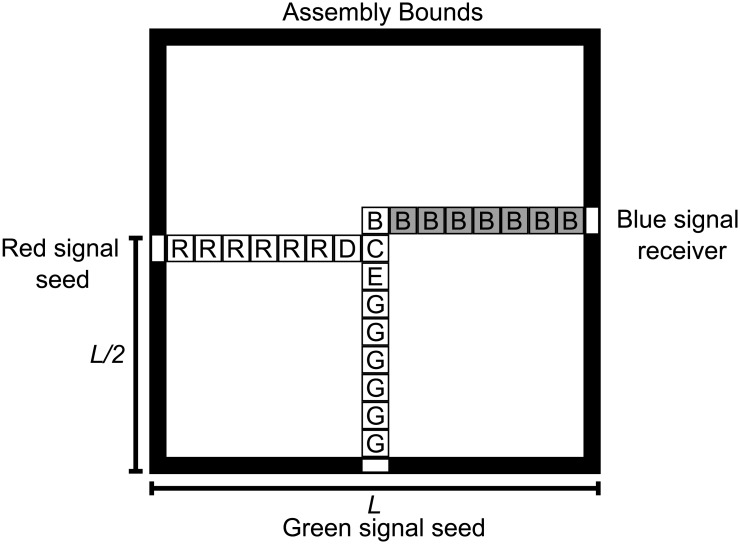
The ideal RGB assembler is designed to grow two signals, one red from site R and one green from site G, toward a point of cooperation C. The seed is an *L* × *L* square perimeter where inputs R and G and output B are to be placed. Cooperation is achieved at the point C, where the signals would “meet”to effect the AND and produce a deterministic signal that is recorded at output B.

The second example, dubbed the RGB assembler (Fig 3), was designed to emphasize issues associated with nondeterminism, cooperative binding, and the effects of finite reaction spaces or numbers of reactants. In a variant aTAM, the assembly space is bounded by a given square seed of side size *L* with a designated point of cooperation at (*L*/2, *L*/2) in the support. This frame is an abstraction that represents the effects of a finite-sized reaction vessel and limited numbers of reactants. The RGB assembler is designed to nondeterministically grow two signals, one red from gate R and one green from gate G, towards a point of cooperation C. If and when C is reached, another signal is produced from C that reports successful cooperation at a certain site B. Minimal cooperation at a single site C is required to nondeterministically produce and record a logical AND signal at an output location B from given seed input signals R and G, all three located on the perimeter of the tiling area, as illustrated in Fig 4. The target set of patterns consists of an assembly successfully implementing the AND gate and the partial assemblies produced by the assembler required to build the circuit, *i.e*. $A_{n}^{\prime }=P_{n}$. (A similar gadget has been used previously in the proof of the Window Movie Lemma [36].) Two assemblers G_*c*_ and G_*e*_ will be compared for efficiency using probabilistic analysis and the Manhattan radius for assembly size.

The first cooperative gate assembler G_*c*_ consists of a tile set for the red and green signals as given in Fig 3, and is designed to operate at *τ* = 2. The blue signal is composed of a hard-coded tile set (with each location accepting only a single, unique tile) that deterministically tiles a path to the receiver B from the point of cooperation C. The second assembler G_*e*_ is identical except that it operates at *τ* = 1, as an implementation of G_*c*_ potentially would in a worst case, experimental setting. In G_*e*_, all strength 2 bonds in the tile set are stable at *τ* = 1.

In order to derive *p* for some assembler, we must determine whether the assembler is capable of producing the target set and the pattern least congruent to any pattern of the same size in the target set. We determine *q* for simple assemblers by counting and finding the relative size of the set of target patterns to the set of error patterns. Table 1 summarizes the results of probabilistic analysis of the two assemblers. The cooperative tile set G_*c*_ probabilistically assembles the target set with *p*_*c*_ ≈ 1/2 and impurity *q*_*c*_, which grows as *O*(*L*). The algorithmic assembly of patterns assures reasonable control of the formation of target patterns. The pattern with least congruence is the assembly sequence that immediately attaches tiles in error to the seed. In this case, there are two congruent tiles and two noncongruent tiles. Growth stops at this point. We determine the impurity *q* by determining the ratio of erroneous patterns to target patterns in the assembled set. In this case, past the point of cooperation C, the size of the target set is just 1. We find the number of error patterns is linear in *L* since there are *L* possible error patterns assembled as a signal grows towards C. In other terms, the longest partial signal (R or G) sets the Manhattan radius and the shorter signal may vary *L* ways.

**Table 1:** Efficiency of a cooperative assembler at temperature *τ* = 2 and its noncooperative implementation at temperature *τ* = 1. The substantial drop in the probability of assembly with respect to the target set and its efficiency show the lack of robustness of the original assembler. Nevertheless, the data shows that some cooperation is better than none for efficiency of the assembler.

Value	Before Cooperation	At Cooperation	Before Blocking	At Block
*p*_*c*_	$\approx \frac{1}{2}$	$\approx \frac{1}{2}$	$\approx \frac{1}{2}$	1
*q*_*c*_	≈ 3	*O*(*L*)	*O*(*L*)	Any *q* > 0
*E*_*c*_	$\approx \frac{1}{6}$	$\Omega (\frac{1}{L})$	$\Omega (\frac{1}{L})$	≈ 0
*p*_*e*_	$\approx \frac{1}{L}$	$\approx \frac{1}{2}$	$\approx \frac{1}{3}$	$\approx \frac{1}{4}$
*q*_*e*_	Ω(*L* ^3^)	Ω(*L* ^2^)	Ω(*L* ^3^)	Ω(*L*)
*E*_*e*_	$O(\frac{1}{L^{4}})$	$O(\frac{1}{L^{2}})$	$O(\frac{1}{L^{3}})$	$O(\frac{1}{L})$

**Fig 5:**
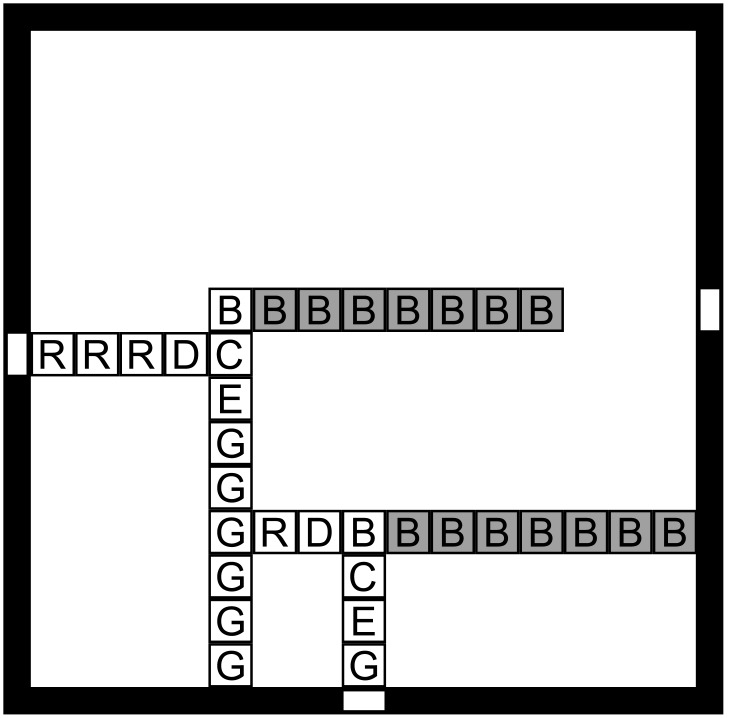
The RGB assembler running in (noncooperative) experimental conditions can produce a variety of error patterns, both before and after the designed point of cooperation. Either the red, green, or blue signal may set the radius (the size of the pattern.) Here, both signals attempt to fake cooperation by nondeterministically attaching a cooperation tile and spawning erroneous color signals. Each possible red and green signal may do so, producing a large quantity of error patterns.

**Fig 6:**
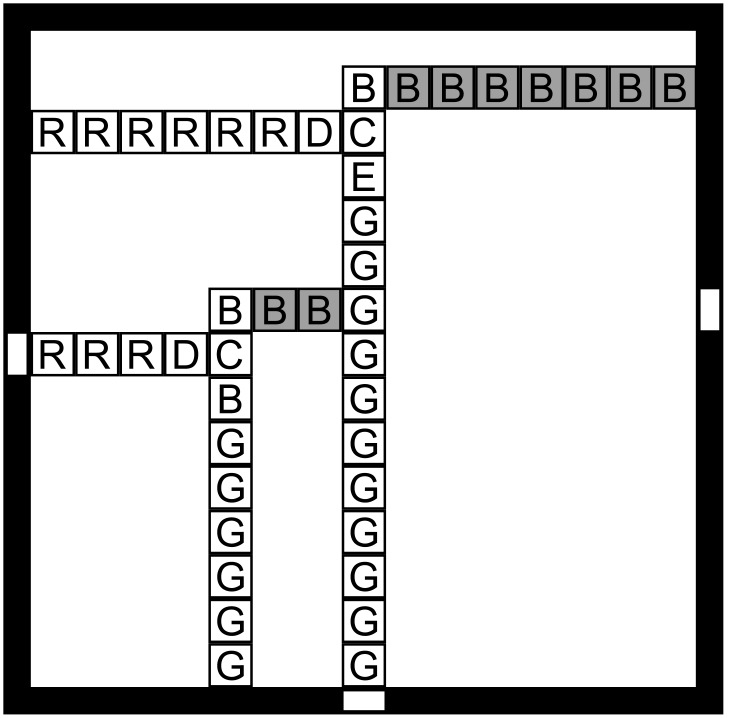
Past the point of cooperation, these phantom signals may or may not block one another, compounding the rate of error patterns.

On the other hand, the noncooperative G_*e*_ (Figs 5 and 6) lacks the control seen under cooperative conditions in G_*c*_. The noncooperative tile set probabilistically assembles the same target set with p_e_ ≈ 1/*L* and impurity *q*_*e*_ that degrades as Ω(*L* ^3^), *i.e*., at least order of *L* ^3^. Noncooperative binding of the C tile in erroneous positions is primarily responsible for the increase in impurity. In this case, we cannot be sure that each signal will only grow a blue signal in the presence of the other signal. At *τ* = 1, the system can “backfill” a phantom signal off a misbound C tile. Thus, the least congruent pattern is that one that immediately fakes cooperation in both and grows multiple phantom signals. The red and green phantom signals compete to block near the origin, producing a pattern with at least *L* erroneous tiles and 2 pattern tiles. Past the point C, the mechanism for misbinding of the cooperative C tile and subsequent nondeterministic growth is also responsible for most of the impurity. We must account not only for red and green phantom signals, but also for phantom blue signals. For each size, there is exactly one target pattern, but the number of erroneous patterns is large. Each pattern failing to cooperate at the point C is in error and may additionally grow red, green, and blue phantom signals. These false signals carve up the available assembly space, producing error patterns at a rate Ω(*L* ^3^).

These numbers are derived for the most favorable target set (one that includes every assembly in any assembly sequence whose supremum is a valid terminal assembly that displays the property of the target set, defined as a perfectly placed blue signal that is generated when both red and green signals are present) and the most noncongruent partial assembly to produce it, as illustrated in Figs 5 and 6. Likewise, the other counts can be estimated as shown in Table 1. This example demonstrates that, as to these target patterns, G_*c*_ does make a more efficient assembler than the noncooperative one. In general, cooperativity is a way to control the negative effects of nondeterminism on efficiency. In fact, since misbinding of the cooperative C tile is the major cause of impurity, even partial experimental achievement of cooperativity is better than none.

By contrast to the previous two examples, our third example shows that strong assembly with perfect efficiency can be achieved at temperature *τ* = 1, but on patterns that do not require cooperation. An example is Meunier’s construction [17] by noncooperative assemblers for producing relatively large sets of 1D patterns for a given bound on the size (number of tile types) of the assembler. This construction shows that these assemblers are capable of assembling reasonably complex infinite families of terminal assemblies, where the complexity of the target pattern is measured by its size relative to the cardinality of the tile set. This holistic metric of complexity, however, does not address the issues of how strongly or efficiently the target set of patterns is being assembled. In [17], the author provides tree grammars for noncooperative tile assembly systems in the 2D planar aTAM. We begin our analysis by determining the target set **P**. In this case, the most natural **P** is the set of terminal assemblies producible by some assembler such that each pattern in the target set has a height of $\frac{5(n+2)}{4}-23$, according to [17]. Since only finite terminal patterns are desired, the target set should also contain promising patterns for the desired finite terminal patterns, as in the previous examples. A small example from [17] illustrates the point with a single-seeded assembler with 38 tile types. Meunier gives the size, measured by the 1D Manhattan diameter, as at most 27, so that **A**_*n*_ = **P**_*n*_ = Φ are empty for *n* > 27. Assembly proceeds from the seed, with tileable sites being occupied in a nondeterministic order by tiles that bind stably with the existing aggregate. By design, it avoids costly nondeterministic bindings which may produce nonpromising patterns or lead to infinite assembly sequences, a potential pitfall in noncooperative systems. Thus, as in the cooperative system, the assembler probabilistically assembles the target set with probability *p* = 1 (all target patterns are produced, and each assembled pattern is exactly some target pattern of the same size.) The impurity is equally easy to derive because it produces only target or promising patterns, therefore the difference set of “junk” patterns is empty and perfect purity and efficiency are attained again. This is partially supported by the clever use of what Meunier calls “caves.” These geometric blocking artifacts constrain the assembly space by forcing new tiles to belong to either the construction of a cave, or the exploration of some previously constructed cave. Eventually, new cave construction ceases and all previously constructed caves are completely explored by the assembler. This halts the system’s growth. Similar to the frame in the RGB example above, the primary advantage of this geometric blocking relates to the impurity of the assembler. By ensuring that the assembler eventually exhausts its assembly space (an example of self-limiting assembly), the assembler avoids a large *q*, which is apparent when **A** is finite. This analysis demonstrates that nontrivial target sets are probabilistically assemblable with perfect purity (any *q* > 0 will do) and arbitrarily large efficiency *E* by noncooperative assembly systems in the aTAM.

The fourth example is derived from a probabilistic analysis of Rothemund’s groundbreaking experimental work in DNA origami assemblers [37]. This example demonstrates an alternative notion of size and the applicability of probabilistic analysis to an experimental system. This assembler forms patterns out of long single strands of DNA “stapled” together by especially designed connecting strands. An alternative notion of size must be used for this probabilistic analysis. The number *n* of *bound* staples in an origami assembly can be used to partition the set of patterns. In [37], the quality of the assembled product is judged from inspection of atomic force microscope (AFM) images shown in supplementary notes S4 and S5. Sampling and AFM may have introduced defects in the patterns, so that the estimates have to be taken with a big grain of salt. Analysis of his fidelity (defined as the number of perfect patterns divided by the total observed in a sampling of the products) can, however, be used to obtain some rough estimate of the strength and efficiency of the assembler (here the assembly protocol.) For this example, there is a single terminal target pattern, ∣**P**_*n*_0__∣ = 1 for the desired number of staples *n*_0_, and the corresponding promising patterns leading to it for *n* < *n*_0_, as before, with all other **P**_*n*_ = Φ for *n* > *n*_0_. We can use the counts of nontarget patterns used in the calculation of his fidelity to estimate *q* based on *n* = *n*_0_ since successful formation of the target pattern implies successful formation of smaller partial assemblies leading to it. The strength *p* is grossly estimated from patterns that are least similar to the target by roughly counting the missing staples. For DNA origami, the *p*’s and *q*’s are shown in Table 2. Better estimates can be obtained from other origami assemblers supported by gel electrophoresis as in [38]. Gels give a good indication of the “junk” obtained at sizes *n* < *n*_0_. The protocol assembles origami squares into “superorigami” structures with a reported yield of at least 95%. Our probabilistic analysis of this origami assembler yields values of *p* closer to 1 and *q* closer to 0 using supplementary figures S8 and S9 from [38] including AFM images depicting folded squares with high congruency and gel electrophoresis showing little partially folded product or “junk”.

**Table 2:** Strength and efficiency of DNA Origami assemblers. Values are roughly estimated based on their Monte Carlo sampling of the assembled products, with *p* being estimated as the smallest fraction of well-formed subpatterns in the target pattern and *q* being estimated as the complement of his fidelity (fraction of correct origamis.) Due to incomplete information, these estimates disagree with the general perception that origami protocols are efficient assemblers.

Pattern Criterion	Square	Rectangle	Star	Smiley
p	0.2	0.5	0.4	0.3
q	≈ 39	≈ 4	≈ 67	≈ 27
E	≈ 0.005	≈ 0.125	≈ 0.005	≈ 0.01

In each of the last three examples, the growth of the assembler is self-limiting or under severe assembly restrictions. Physically or metaphorically, these assemblers work inside of a finite assembly space as though on a prepared substrate or in a finite test tube. Meunier’s noncooperative assemblers build walls as they grow the pattern, creating and then exploring finite caves in a process that eventually exhausts all possible future construction. The RGB assembler grows in a tiling space bounded by a given seed. Rothemund’s origami acts on a single finite strand (rather than the tiling notion of tile types in unbounded number.) In these systems, each new tile or staple constrains the space of possible future attachments, which allows for high efficiency. In the absence of these provisos, pumpable paths of tiles can yield extremely inefficient assemblers.

These examples show that the two concepts of assembly strength and assembly purity of an assembler with respect to a target set can vary independently, except for the case of *p* = 1. When *p* = 1, the assembler must assemble all target patterns of every size, every assembled pattern must be a target pattern, and there is no room for impurity. However, when *p* < 1, assembled patterns may largely resemble target patterns but they still may be either target or junk patterns. A final family of examples described in detail in Appendix A exhibit *deterministic* assemblers that assemble patterns with high probability close to but strictly smaller than 1 and still with perfect purity, for the set of target patterns assembled by *nondeterministic* and more complex assemblers with perfect strength and purity. These examples also illustrate how probabilistic assembly suggests a more general concept of simulation of an assembler by another that does not involved direct simulation of the dynamic process or kinetics of the original system. Because of the technical concepts involved, these systems are fully described in Appendix A.

### General Results on Probabilistic Analysis

This section presents some general results that give a sense of the nature of probabilistic analysis, as well as its capabilities. One of the primary questions alluded to above is the relationship of the observable strength *p* and impurity *q* to the probability of a pattern being assembled, *i.e*., the worst-case for the ratios *p*_*n*_ = ∣**A**_*n*_∩**P**_*n*_∣/∣**A**_*n*_∣, which is much harder to observe in an experimental setting or for very complex assemblers.

**Theorem 1**

*If a set of patterns* **P** *is probabilistically assemblable with probability p, impurity q and efficiency* $E=\frac{p}{q}$ *by a monotonic pattern assembler G , then*

(a) *target patterns are assembled with positive probability at least* $\frac{Kp}{p+q}=\frac{KE}{1+E}>0$, *where K is the minimum positive probability of attachment of any single tile in the assembler; promising patterns are assembled with positive probability at least* $\frac{p}{p+q}=\frac{E}{1+E}>0$;
(b) *nontarget (erroneous + nontarget promising) patterns are assemblable with probability at most* $1-\frac{Kp}{p+q}=\frac{(1-K)p+q}{p+q}=\frac{1+(1-K)E}{1+E}$.
(c) *With respect to all assemblies, erroneous patterns are assembled with a complementary probability to that of promising patterns and within the interval* $\left[0,\frac{1}{1+E}\right]$.

*Proof*.

Let *p*_*n*_, $a_{n}^{\prime }$, and *e*_*n*_ denote the probabilities of target patterns, promising patterns, and erroneous patterns of size *n* being assembled, respectively. For every size *n*, the probability of a target pattern being assembled is given by $p_{n}:=\frac{|A_{n}\cap P_{n}|}{|A_{n}|}$. The hypotheses thus guarantee that $\frac{|A_{n}\cap P_{n}|}{|P_{n}|}\geq p$, so that $p_{n}=\frac{|A_{n}\cap P_{n}|}{|A_{n}|}=\frac{|A_{n}\cap P_{n}|}{|P_{n}|}\frac{|P_{n}|}{|A_{n}|}\geq p\frac{|P_{n}|}{|A_{n}|}$, and that $e_{n}=1-a_{n}^{\prime }=\frac{|A_{n}-A_{n}^{\prime }|}{|A_{n}|}=1-\frac{|A_{n}^{\prime }|}{|A_{n}|}\leq q\frac{|P_{n}|}{|A_{n}|}$, *i.e*., $1-a_{n}^{\prime }\leq q\frac{|P_{n}|}{|A_{n}|}\leq q\frac{p_{n}}{p}\leq \frac{p_{n}}{p}\frac{|A_{n}^{\prime }|}{|A_{n}|}=\frac{q}{p}a_{n}^{\prime }$ because $p_{n}\leq \frac{|A_{n}^{\prime }|}{|A_{n}|}=a_{n}^{\prime }$ since $A_{n}\cap P_{n}\subseteq A_{n}^{\prime }$. Solving for $a_{n}^{\prime }$ we obtain that $a_{n}^{\prime }\geq \frac{1}{1+\frac{q}{p}}=\frac{p}{p+q}>0$, *i.e*., the inequality in (*a*) holds for promising patterns. Now, since the assembler is monotonic, every target pattern *x* is obtained from a nontarget promising pattern *a* in one step (as the last in a chain of only promising patterns), and then the probability of *x* is given by the conditional probability

$$p\left(x\right)=p\left(x|a\right)p\left(a\right)\geq K\frac{p}{p+q}\;,$$

where *K* is the minimum probability of attachment of a single tile (here of the tile added to *a* to obtain *x*.) Therefore, the inequality in (*a*) holds for target patterns as well. Statement (*c*) is straightforward from (*a*) and the fact that $1-\frac{E}{1+E}=\frac{1}{1+E}$. For inequality (*b*), The nontarget patterns are $E_{n}\cup (A_{n}^{\prime }-\left(A_{n}\cap P_{n}\right)=A_{n}-\left(A_{n}\cap P_{n}\right)$. Therefore, $e_{n}+a_{n}^{\prime }-p_{n}=\frac{|A_{n}-\left(A_{n}\cap P_{n}\right)|}{|A_{n}|}=\frac{|A_{n}\left|-\right|A_{n}\cap P_{n}|}{|A_{n}|}=1-\frac{|A_{n}\cap P_{n}|}{|A_{n}|}\leq 1-K\frac{p}{p+q}=\frac{1+(1-K)E}{1+E}\;\;.$

In manufacturing, the yield refers to the fraction of error-free target, in our notation ∣**A**_*n*_∩**P**_*n*_∣/∣**A**_*n*_∣ = (*KE*)/(*E*+1), while in chemistry it is described as the fraction of target product obtained of the theoretical optimum.

Another important question concerns information about the dynamic behavior of an arbitrary assembler based solely on estimates of its strength and efficiency. The next results shows that a taxonomy of assemblers is indeed possible based on these parameters alone.

**Theorem 2**

*If a set of patterns* **P** *is efficiently probabilistically assemblable by a pattern assembler G , then one of the following three alternatives must hold*:

(a) *either the rate at which (erroneous + nontarget promising) are assembled eventually stabilizes at a constant value;*
(b) *or the rate at which (erroneous + nontarget promising) are assembled switches in value in an alternating fashion;*
(c) *or the rate at which (erroneous + nontarget promising) are assembled is at least exponential*, *i.e*. |*A_n_k__*| = Ω(*α^n_k_^*) *or* $|A_{n_{k}}^{\prime }|=\Omega \left(\beta^{n_{k}}\right)$ *for infinitely many n*_*k*_, *for some constant rate α* > 1 *or some constant rate β* > 1.

The proof requires the following concepts in real analysis in ordinary Euclidean space. A limit point of a given a sequence of real numbers {*a*_*n*_}_*n*_ is the limit of some converging subsequence {*a*_*n*_*j*__}. Thus a converging sequence has only one limit point (its limit), but nonconvergent sequences such as {(−1)^*n*^} can have more than one limit point (such as ±1.) The well known Bolzano-Weierstrass Theorem says that a bounded sequence of points in a real closed interval always has at least one limit point. This nonempty set of limit points for a sequence {*a*_*n*_}_*n*_ will be denoted *Lim*({*a*_*n*_}).

*Proof*.

Let *ρ*_*n*_ and *ν*_*n*_ denote the infinite sequences defined by $\rho_{n}:=\frac{|A_{n}^{\prime }|}{|A_{n}|}$ and $\nu_{n}:=\frac{|A_{n}-A_{n}^{\prime }|}{|A_{n}|}$ for the corresponding sets of assembly for promising and erroneous patterns, as defined above. From Theorem 2(c), there follows that $0\leq \rho_{n}+\nu_{n}<1-\frac{Kp}{p+q}$ for every pattern size *n* ≥ 0, so these sequences are bounded, and *Lim*(*ρ*_*n*_) and *Lim*(*ν*_*n*_) are nonempty sets, by the Bolzano-Weierstrass Theorem.

If *Lim*(*ρ*_*n*_) and *Lim*(*ν*_*n*_) consist of single elements, clearly condition (*a*) holds. Otherwise, let *ρ* and *ν* be the respective supremum (the smallest upper bound) of their nonempty limit sets. Only one of two alternatives hold, either *ρ* = *ν* or *ρ* ≠ *ν*. If *ρ* = *ν*, there must exist infinitely many cases in which *ρ*_*n*_ ≥ *ν*_*n*_ holds and infinitely many cases in which *ρ*_*n*_ ≤ *ν*_*n*_ holds. Therefore there will be infinitely many intervals during which each of them predominates over the other, as stated in (*b*).

Otherwise, *ρ* ≠ *ν* and one type of patterns (promising or faulty) eventually dominates. Given a promising or faulty pattern *x* of size *n*, let the *context* of an assembled pattern refer to a site where one tile may stably attach according to the given assembler and let the *boundary* be the set of all contexts for an assembly of size *n*. If *x* is faulty, every possible extension of the pattern will remain faulty, regardless of the boundary. Let *α*_*n*_(*x*) be the number of all such possible attachments. If *x* is promising, only a certain number of attachments *β*_*n*_(*x*) are not erroneous. The number of erroneous and promising attachments are complementary to the set of all possible attachments. Let *α*_*n*_ and *β*_*n*_ be the minimum of *α*_*n*_(*x*) and *β*_*n*_(*x*) over all patterns *x* of size *n* and let *α* and *β* be the infimum (greatest lower bound) of all *α*_*n*_ > 0 and *β*_*n*_ > 0 over all *n* > 0. Since *ρ* ≠ *ν*, at least one of them must be positive and, in fact, greater than 1 for infinitely many *n*. Therefore *ρ*_*n*_ ≥ *cα* ^*n*^ or *ν*_*n*_ ≥ *cβ* ^*n*^ for infinitely many *n*.

## Discussion and Conclusions

Self-assembly, whether used *in vitro* to construct nanoscale structures or as exhibited in nature, is fundamentally a stochastic process that is subject to random disorder. The primary problem addressed in this paper is how to quantify the efficiency of a pattern assembler (a model of self-assembly, its implementation *in vitro*, or any other type of self-controlled assembly system) in producing a given target set of patterns, even if nondeterministically and capable of producing nontarget (junk) patterns. This question is analogous to the well-known problem of assessing the computational complexity of an algorithm (possibly randomized) solving a computational problem. A meaningful answer to this question likewise requires prior identification of the target set of patterns to be assembled, a proper definition of what it means to assemble a pattern and a metric to measure the size of patterns, in addition to a definition of efficiency of an assembler. A solution to the problem will likewise provide a way to compare the relative efficiency of two assemblers and a concept of the best assembler for the target set among a group of alternatives. A fairly general definition of probabilistic pattern assembly has been presented, along with an appropriate definition of efficiency of an assembler. The conditions require, not only that the assembler uniformly produce a certain fixed fraction *p* of the target patterns for every size *n*, but that it have a good “idea” of the target set, *i.e*., that every assembled pattern of size *n* share a fraction *p* of some target pattern of the same size. The assembler is defined to have impurity *q* if it uniformly produces at most a factor *q* of nonpromising (junk) patterns for every size *n* with respect to the size of target patterns. The corresponding probabilistic analyses of typical assemblers in the self-assembly literature show that cooperative aTAM assemblers (at temperature *τ* = 2) produce families of patterns deterministically with probability *p* = 1 and arbitrarily large efficiency (with arbitrarily small *q* > 0), primarily because they only assemble finite sets or unique patterns. Their efficiency, however, is substantially reduced when compared to the same assemblers run noncooperatively (at temperature *τ* = 1), *i.e*., they are not likely to be very robust assemblers experimentally. When assembling certain classes of infinite sets of 1D target patterns, nondeterministic assemblers can also perform at probability *p* = 1 and with arbitrarily large efficiency.

Examples also show that this concept of probabilistic assembly is valid and useful to compare the performance of different assembly systems on the same set of target patterns. Even if an ideal deterministic and cooperative assemblers is efficient, the corresponding assembler in which nondeterministic and noncooperative bonds are possible may, in general, be not efficient. Nevertheless, achieving any level of cooperation improves the efficiency over none at all. This result reinforces models like the kTAM [21], and motivates a search for mechanisms of cooperativity that are more experimentally accessible. When noncooperative assembly is appropriate or when cooperativity is not required, efficient assemblers are possible, and in fact, noncooperative assemblers inherently achieve them. Thus, because they are more likely to produce good experimental results, applications that do not require cooperativity are worth exploring in detail in a search for more efficient assemblers. Probabilistic analysis produces results that are consistent with current intuition in algorithmic assembly models, while also providing some insights into the experimental feasibility of existing models.

Some general results have been established as well. First, every assembler assembling a target with strength *p* and (im)purity *q* must uniformly produce every target pattern with probability at least $\frac{E}{E+1}$, and erroneous patterns at a rate bounded by $\frac{KE}{1+E}$, where $E=\frac{p}{q}$ is the efficiency of the assembler and *K* is the minimum positive probability of attachment of a tile in the assembler. This framework and its results are quite general and apply to arbitrary assemblers, including the aTAM and experimental systems, as long as the system is monotonic, *i.e*., the assembly process can only enlarge the size of an assembly. The general results also demonstrate the utility of the model to examine or reason about efficiency or simulation of self-assembly without recourse to the details of the assembly model under consideration.

A number of questions are suggested by this probabilistic type of analysis of pattern formation. First, the standard measure of complexity of an aTAM assembler has been the size of the tile set used to produce the target set. The efficiency is measured by the size of the assembled patterns relative to the size of the tile set. The notion of efficiency *E* seems to bear no relation to the size of the tile set in the assembler. Is there any relationship between the two concepts? For example, it appears plausible that an upper and/or lower bound could be placed on the size of an optimal tile set necessary to simulate an assembler of a given strength *p* and efficiency $E=\frac{p}{q}$.

Second, the efficiency *E* provides a combined measure of strength and purity, a measure of the tradeoff between attempting to produce the most patterns in the target set while simultaneously producing the minimum amount of junk patterns. A larger efficiency indicates better strength and/or more purity (a smaller fraction of junk with respect to the set of target patterns.) By Theorem 1, efficiency of *E* > 1 guarantees that the probability of producing a promising pattern is at least *K*/2, as can be readily verified. Is *E* = 1 the appropriate threshold of efficiency that characterizes practical assemblers useful in the laboratory from assemblers that are not? Or, should one replace *p* and *q* by analogous functions of size *n* and rather require that the ratio $\frac{p_{n}}{q_{n}}$ grow at some polynomial rate *f*(∣**P**_*n*_∣) in the number of target patterns ∣**P**_*n*_∣? Regardless of the value, the related problem of then characterizing efficiently probabilistically assemblable sets of patterns according to an appropriate threshold appears to be an interesting and difficult problem.

Third, the efficiency *E* thus provides a way to holistically compare two assemblers of a target set for quality and, therefore, implicitly provides a criterion for choosing a best assembler for a given target set, namely one with unbeatable efficiency. How does this concept of optimality relate to the size of the most efficient tile set that assembles the given set probabilistically? At first glance, there appears to be no connection even for strict assembly (*p* = 1 and perfect efficiency), but one cannot completely rule out that a thrifty tile set may need to produce some junk to assemble the target patterns.

A fourth question posed by this approach is the exact power of nondeterministic assembly with respect to locally deterministic assembly. The examples show that running a cooperative assembler at temperature *τ* = 1 substantially decreases its efficiency due to “errors” in the cooperative assembly that increase the nondeterministic choices in the assembly process. These examples show that there is a difference, but others in Appendix A also show that in some cases one might be able to “determinize” the assembly at the price of a smaller strength. Characterizing probabilistically determinizable assemblers is analogous to the corresponding complexity question and also appears to be a difficult problem.

## Appendix

An important question in self-assembly is the power that nondeterministic assembly affords relative to deterministic models. Some insight into this question can now be obtained through probabilistic analysis because it allows a new way to compare the assembly power of different assemblers without resorting to direct dynamic simulation. The two examples below compare a particular kind of nondeterministic assembly system, namely probabilistic assemblers, to deterministic counterparts.

The assemblers are tile assembly implementations of cellular automata (CA). A CA is a computational model of a physical system defined on a homogeneous lattice, such as integer lattices in Euclidean spaces, by a set of rules that specify the state transitions of a discrete computational element at every node. Starting from a given seed as in the aTAM, a typical node/processor *i* synchronously updates its state to a value *δ*(*x*_*i*−1_, *x*_*i*_, *x*_*i*+1_), according to a common local rule *δ* based on the current state values of its immediate neighbors *i* − 1, *i* (itself) and *i* + 1, for rules of radius *r* = 1. These rules, called elementary cellular automata (ECA), define another type of assemblers of 2D patterns that describe their space-time evolution from an initial seed. There are 256 such ECA, each of which can be described by 8-bit number between 0 and 255, so that the binary expression gives in its *k* ^*th*^ bit-value the *δ*(*a*, *b*, *c*) for the local 3-bit blocks *abc* encoding *k* in binary. These rules produce a variety of interesting space-time behaviors that have been extensively studied and are fairly well understood. Further details about cellular automata can be found in [39, 40]. ECA assemblers are the simplest examples of deterministic assemblers producing infinite sets of complex and interesting target patterns, since they are also computation universal. The appropriate notion of size for these sets is naturally the height *n* of the pattern, *i.e*., the time it has taken the ECA to produce its assembly from the initial seed by the deterministic rule *δ*.

There is a deterministic cooperative (temperature *τ* = 2) tile assembler associated with every ECA defined as follows.

1. Create one tile out of every 3 × 3 chunk of CA space time representing the transitions $a_{i}^{\prime }=\delta \left(a_{i-1},a_{i},a_{i+1}\right)$ and $a_{i}^{\prime \prime }=\delta \left(a_{i-1}^{\prime },a_{i}^{\prime },a_{i+1}^{\prime }\right)$;
2. Label edges (with dashes) and corners (with dark quarter circles) whenever the value at that location is 1 (active);
3. *a*_*i*−1_, *a*_*i*_, *a*_*i*+1_ are located at the W, NW and N locations, respectively, at time *t* (top line);
4. The value $a_{i}^{\prime }=\delta \left(a_{i-1},a_{i},a_{i+1}\right)$ and possible flanking sites (4 values for 4 tiles) $\left(a_{i-1}^{\prime },a_{i}^{\prime },a_{i+1}^{\prime }\right)$ are located at W, C and E, respectively, at time *t*+1 (middle line); if $a_{i}^{\prime }=1$, then W and E are labeled;
5. $a_{i}^{\prime \prime }=\delta \left(a_{i-1}^{\prime },a_{i}^{\prime },a_{i+1}^{\prime }\right)$ is located at the SE location at time *t*+2 (bottom line)
6. Create a new binding domain for each 3 bit binary number “read” from the edge of each tile

**Fig 7:**
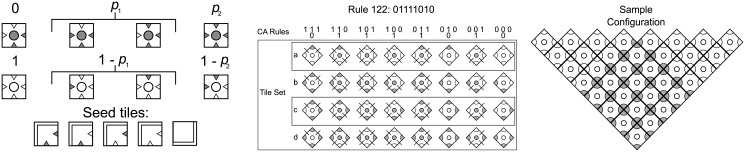
Seed tiles and transition probabilities of probabilistic cellular automaton DKCA assemblers (Left). The tile set (Center) for ECA rule 122 uses tile concentrations and nondeterminism to weakly assemble a variety of patterns (without spontaneous generation) from random seeds, as illustrated on the Right.

In the pattern of its glue types and symbols assigned to tiles, this tile set (Fig 7)(Left) assembles with probability *p* = 1 the target set of finite space-time 2D patterns generated by the original 1D ECA, on a given seed of arbitrary size. This tile set simulates the original ECA in the following sense.

**Definition 6**. *An assembler G probabilistically simulates assembler* ℋ *if* G *probabilistically assembles* **A**(ℋ), *the set of patterns produced by* ℋ, *with some probability p > 0.* G *simulates* ℋ *if* G *probabilistically simulates* ℋ *with p = 1*.

**Fig 8:**
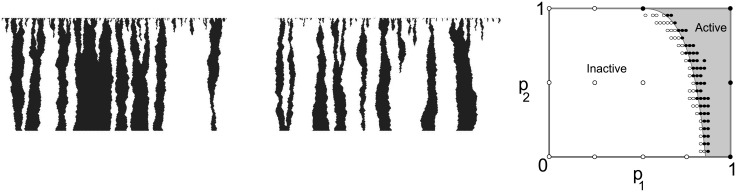
DKCA patterns generated from the same seed can be wildly different (Left/Center). The DKCA phase diagram (Right) describes the observable behavior of the DKCA across a spectrum of probabilities *p*_1_ and *p*_2_, with typical non/percolating behavior of an ECA assembler (shown as filled/empty circles, respectively) consistent with the phase-transition DKCA behavior overall (the thresholding curve separates percolating from nonpercolating CA regions.)

A second example is the Domany-Kinzel Cellular Automaton (DKCA)[41], a 2D probabilistic assembler defined by a CA on binary states of radius *r* = 1 whose transitions occur nondeterministically with probabilities given by

$$p\left[1|0,0\right]=0,\;p_{1}=p\left[1|0,1\right]=p\left[1|1,0\right],\;p_{2}=p\left[1|1,1\right]\;$$

where 0/1 indicates an inactive/active (zero/nonzero) site and *p*[0∣⋅,⋅] = 1 − *p*[1∣⋅,⋅]. Starting from a given initial condition *s* ≡ *x* ^0^ at time *n* = 0, simultaneous application of the transition rules defined by *δ* with the given probabilities produces another configuration *x* ^*t*^ at time *t* = 1, and iteration over time produces again 2D binary patterns of unbounded size by a random process. The DKCA is an abstract model of directed percolation, a phenomenon exhibited by directed spreading processes [41]. An important result in directed percolation theory is the existence of a phase transition from inactive (state 0) to active (state 1) global states as the combination of *p*_1_ and *p*_2_ across a certain critical value on their way to 1, at which point the 2D pattern generated is surely guaranteed to be percolating, *i.e*. locations in the seed are connected by paths of active sites to sites in the last row. An example of a percolating configuration is shown in Fig 8(a) and 8(b) and the full active region is shown in the phase diagram on the right. We denote with $P_{G}^{Perc}$ the set of percolating patterns generated by a CA assembler G .

A tiling system can again be designed that reproduces the space-time history of the DKCA, when operating at temperature *τ* = 2. In the mapping from DKCA to assembly systems (Fig 7), Center and Right), a tile is constructed to represent all possible probabilistic transitions for the DKCA. The North and West edges (called input edges) have strength 1 glues and are marked 1 or 0 based on the input to the rule. The center of the tile is marked 0 (inactive) or 1 (active) based on the output. The East and South edges are marked with the label of each tile. For each tile in the tile set, a concentration is assigned such that the probability of attachment to an aggregate corresponds to the DKCA parameters *p*_1_ and *p*_2_. For the weak assembly [35] of the entire space-time history, there exists a concentration function *χ* which describes the relative concentrations of each tile to other tiles. With this mapping below, these tile assembly systems are parametrized by the DKCA parameters *p*_1_ and *p*_2_ as well.

It is straightforward to verify that these constructions guarantee that the two assemblers (the DKCA and the ECA tile sets) simulate each other with probability *p* = 1, according to Definition 6. The assembler is maximally nondeterministic since, as usual, all tiles are available in saturation amounts. This observation implies the following result.

**Theorem 3**

*The set of target patterns* **P**_*DKCA(p_1_,p_2_)*_ *is strongly probabilistically assemblable with probability p = 1 and perfect efficiency by the corresponding tile set described above for an arbitrary DKCA(p_1_,p_2_)*.

To address the question above, we are particularly interested in a target set of patterns assembled by this DKCA(*p*_1_, *p*_2_) tile set as an example to test whether certain nondeterministic pattern assemblers can be found probabilistically equivalent to *deterministic* assemblers and if so, what exactly is the tradeoff involved in strength and efficiency, as defined in 5. This is in fact the case for several DKCA tile assemblers described next. The height of the pattern (corresponding to the time taken to generate it) is used as the appropriate notion of size.

**Theorem 4**

*The target set of percolating configurations* $P_{DKCA(p_{1},p_{2})}^{Perc}$ *assembled by certain percolating* DKCA(*p*_1_, *p*_2_) *can be probabilistically assembled by a deterministic ECA tile assembly system with high probability, but cannot be probabilistically assembled with probability p = 1*.

*Proof*. We consider only ECA without the spontaneous generation of active sites (labeled 1), *i.e*., cells in the CA will remain inactive if the left and right neighbors are inactive, or, equivalently, such a cell will become active with probability 0 as a probabilistic CA. For a given DKCA(*p*_1_, *p*_2_), the target set of percolating patterns $P_{DKCA(p_{1},p_{2})}^{perc}$ includes a wide variety of cluster shapes and sizes. From the construction of the assemblers, an ECA assembler essentially reproduces the tiling coding of the space-time from any seed and therefore only produces one pattern of a given height. In order to simulate a DKCA assembler, the transition rules of an ECA assembler have to be applied with the same probabilities (*p*_1_, *p*_2_). These probabilities are *not* identical to the probabilities in the transition rules of the ECA because its space-time generally has a structure that may eventually bias the applicable rules as the pattern is generated. However, they can be empirically estimated from simulations *in silico* of the ECA from random initial conditions. These restrictions, coupled with the fact that each seed only produces one pattern of a given size (height) *n*, imply that an ECA assembler can, in general, only reproduce a fraction of the set of percolating patterns generated by a DKCA at best. The ECA considered do not blanket the phase space evenly, as shown by simulation results *in silico* on the Right in Fig 8. However, for certain particular empirical values *p*_1_ and *p*_2_, the ECA can probabilistically assemble the set of percolating patterns produced by the DKCA(*p*_1_, *p*_2_) with a high probability (*p* > 0.9 in our simulations), as described next. Stochasticity in the tiling comes from the random initial conditions.

The strength *p* of the simulation was estimated experimentally as follows. For each candidate ECA, 100 tiling simulations at size 300 were performed. Each tiling was run to completion (filling the simulation space as defined by the seed) at temperature *τ* = 2 with no chance of error or vacancy. Likewise, the corresponding DKCA for the empirical probabilities (*p*_1_, *p*_2_) were simulated. By counting the actual use of each rule in the space-time history of the ECA over many runs and then determining the number of active children and inactive children generated by the appropriate rules, an estimate can be given of which DKCA is producing the patterns. The space-times of the two assemblers were tested for percolation and thus how likely the two assemblers agree on the percolation property. Percolation in a finite assembly was determined by the presence of a spanning cluster of active sites that is incident on both the seed and the output row. An ECA assembler simulates the DKCA when it produces a set of patterns **A** that probabilistically assembles the target **P** ^*perc*^ with one of two thresholds, *p* > 0.9 and *p* > 0.94. The resulting phase space diagram for the tile set reproduced the original DKCA phase space diagram [41] at the sample points, as shown on the Right in (Fig 8). This shows that the ECA assembler generates a nonzero fraction of the DKCA patterns, and that it does not produce all of them *i.e*., *p* < 1.

Thus, these examples exhibit deterministic assemblers (ECA) that assemble patterns with high probability close to but strictly smaller than 1 and still with perfect purity, for the set of target patterns assembled by nondeterministic and more complex assemblers (DKCA) with perfect strength and purity. They also illustrate how probabilistic assembly suggests a more general concept of simulation of an assembler by another that does not involve direct simulation of the dynamic process or kinetics of the original system.
